# Supplementary material for: Selinexor Sensitizes TRAIL-R2-Positive TNBC Cells to the Activity of TRAIL-R2xCD3 Bispecific Antibody
Source: Cells. 2020 Oct 2;9(10):2231. doi: 10.3390/cells9102231 (PMC7599683; doi:10.3390/cells9102231)
Supplement: Supplementary file 1 [file cells-09-02231-s001.pdf]

cells

MDPI

Supplementary files

Table 1. Concentrations of selinexor required to inhibit cell growth by 50% (IC_{50}) as determined by MTS assay after 72 h continuous exposure. Data represent the mean±SD of at least three independent experiments.

Cell Line	Selinexor IC50 (µM)
MDA-MB-468	0.03 ± 0.02
SUM-159	0.10 ± 0.04
MDA-MB-231	0.03 ± 0.05
MS-186	0.09 ± 0.01
MCF-10A	>10

Table 2. Combination index values for the selinexor -TRAIL-R2xCD3 BsAb combined treatment in SUM-159 cell line. [a]Combination index as determined by the median effect method using CompuSyn Software: CI < 1 indicates synergy, CI = 1 indicates additivity, and CI > 1 indicates antagonism.

Selinexor / BsAb (µM / µg/mL)	CI[a]
0.1/1	**0.80**
0.1/0.5	**0.20**
0.1/0.25	2.40
0.1/0.125	1.50

Supplementary figure 1

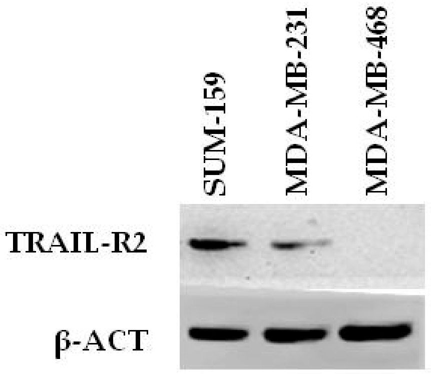

Supplementary figure 1. TRAIL-R2 expression of TNBC cell lines. Western immunoblotting showing the expression of TRAIL-R2 in SUM-159, MDA-MB-231 and MDA-MB-468.

Supplementary figure 2

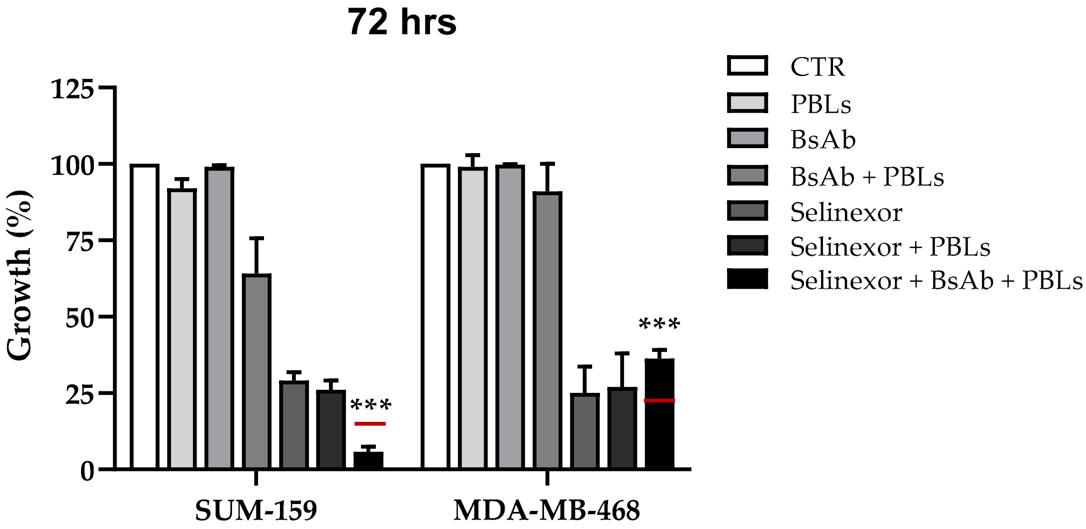

Supplementary Figure 2. SUM-159 and MDA-MB-468 cells were exposed for 24h to selinexor (1.0μM and 0.2μM, respectively) and then treated with 0.5 μg/mL TRAIL-R2xCD3 BsAb + PBLs (E:T ratio = 5:1) for additional 72h. The cytotoxic effect of individual and combined treatments was assessed by MTS assay at the indicated time points and data are expressed as percentage values of growth in treated cells compared to control (cells exposed to 0.01% DMSO). Bars represent the mean ±SD of three independent experiments. PBLs alone, BsAb alone and selinexor+PBLs were used as control. Red lines represent the expected additive effect of the combination, calculated as the product of the effects of the individual drugs, according to the method of Kern et al.[25]. ***P<0.001.
